# Modelling MOG antibody-associated disorder and neuromyelitis optica spectrum disorder in animal models: Spinal cord manifestations

**DOI:** 10.1016/j.msard.2023.104892

**Published:** 2023-07-17

**Authors:** Jana Remlinger, Maud Bagnoud, Ivo Meli, Marine Massy, Christopher Linington, Andrew Chan, Jeffrey L. Bennett, Robert Hoepner, Volker Enzmann, Anke Salmen

**Affiliations:** a Department of Neurology, Inselspital, Bern University Hospital and Department for BioMedical Research (DBMR), University of Bern, Bern, 3010, Switzerland; b Graduate School for Cellular and Biomedical Sciences, University of Bern, Bern, 3010, Switzerland; c Institute of Infection, Immunity and Inflammation, University of Glasgow, Glasgow, G12 8TA, UK; d Departments of Neurology and Ophthalmology, Programs in Neuroscience and Immunology, University of Colorado Anschutz Medical Campus, Aurora, CO, 80045, United States of America; e Department of Ophthalmology, Inselspital, Bern University Hospital and Department for BioMedical Research (DBMR), University of Bern, Bern, 3010, Switzerland

**Keywords:** MOG antibody-associated disorder, Neuromyelitis optica spectrum disorder, MOG-IgG, AQP4-IgG, Experimental autoimmune encephalomyelitis

## Abstract

Antibodies to myelin oligodendrocyte glycoprotein (MOG-IgG) or aquaporin 4 (AQP4-IgG) are associated with CNS inflammatory disorders. We directly compared MOG_35–55_-induced experimental autoimmune encephalomyelitis exacerbated by MOG- and AQP4-IgG (versus isotype IgG, Iso-IgG). Disease severity was highest after MOG-IgG application. MOG- and AQP4-IgG administration increased disease incidence compared to Iso-IgG. Inflammatory lesions appeared earlier and with distinct localizations after AQP4-IgG administration. AQP4 intensity was more reduced after AQP4- than MOG-IgG administration at acute disease phase. The described models are suitable for comparative analyses of pathological features associated with MOG- and AQP4-IgG and the investigation of therapeutic interventions.

## 1. Introduction

Neuromyelitis optica spectrum disorder (NMOSD) and myelin oligodendrocyte glycoprotein antibody (MOG-IgG)-associated disorder (MOGAD) are antibody-mediated inflammatory disorders of the CNS distinct from multiple sclerosis (MS) (Jarius et al., 2018; Lennon et al., 2005; Wingerchuk et al., 2015).

Pathogenic autoantibodies against the water channel aquaporin 4 (AQP4-IgG) in NMOSD and MOG-IgG in MOGAD, respectively, target different CNS proteins (Spadaro et al., 2016; Zamvil and Slavin, 2015). MOG-IgG is directed against MOG, a myelin protein, and results in demyelination, whereas AQP4-IgG leads to primary astrocyte damage with secondary myelinolysis and neurodegeneration. Even though inflammatory cell infiltration and demyelination are hallmarks of both diseases, pathophysiological differences are observed (Lopez et al., 2021; Spadaro et al., 2018; Zamvil and Slavin, 2015). Immunopathological mechanisms of astrocytopathy and demyelination associated with antibodies against AQP4 in the CNS parenchyma include complement-dependent cytotoxicity (CDC) and antibody-dependent cell cytotoxicity (ADCC) (Ratelade et al., 2013; Ratelade et al., 2012). In vitro investigation of MOGAD patient sera showed surface mobilization of CD107a by natural killer (NK) cells illustrating a capacity for driving NK-mediated cytotoxic activity (Brilot et al., 2009).

The pathogenicity of antibodies against MOG (Lassmann et al., 1988; Linington et al., 1988) and AQP4 (Bennett et al., 2009; Bradl et al., 2009) was shown using variants of rat experimental autoimmune encephalomyelitis (EAE). Antibodies may enter the CNS provided the blood brain barrier (BBB) is breached by underlying neuroinflammation as demonstrated before (Challa et al., 2013; Saini et al., 2013). Thereafter, they bind target structures, fix and activate complement and induce complement-independent mechanisms like ADCC (Bradl and Lassmann, 2016; Bradl et al., 2009). Species differences may be important to account for as data on complement activation by MOG-IgG and AQP4-IgG are sparse, differ in murine and rat models and IgG1 antibodies seem to poorly activate mouse C1q (Ratelade and Verkman, 2014; Sörman et al., 2014).

We directly compared antibody- and non-antibody-mediated murine EAE models to investigate spinal cord manifestations associated with antibodies against MOG, AQP4 or isotype antibodies (Iso-IgG) at different stages of the disease.

## 2. Material and methods

### 2.1. Experimental design

**Fig. 1.**
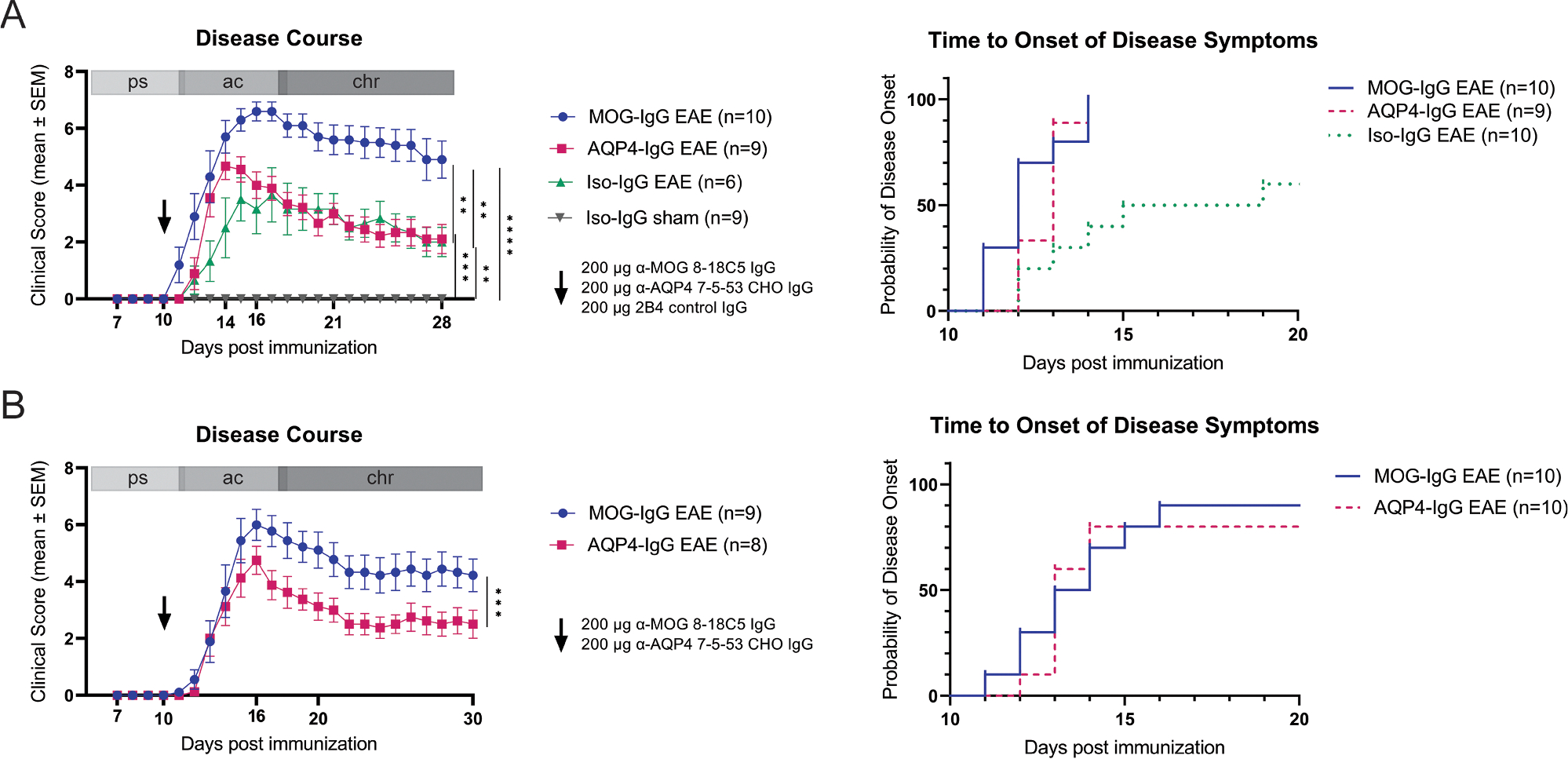
Disease severity and time to onset of disease symptoms analysis of EAE with administration of MOG-, AQP4- or Iso-IgG and sham-immunized controls. (A) EAE disease augmentation with MOG-IgG (200 μg, i.v., blue circles), AQP4-IgG (200 μg, IV, red squares) or Iso-IgG (200 μg, i.v., green triangles) on 10 dpi. Administration of Iso-IgG (200 μg, i.v., 10 dpi) to CFA sham-immunized animals (gray triangles). Mobility impairment was scored daily on a 10-point scale. Two independent experiments. Kruskal-Wallis test. Evaluation of time to disease onset by log-rank (Mantel-Cox) test. (B) Experiment showing EAE disease augmentation with MOG-IgG (200 μg, i.v., blue circles) and AQP4-IgG (200 μg, i.v., red squares) only on 10 dpi. One experiment. Mann-Whitney test. ** *p* < 0.01, *** *p* < 0.001, **** *p* < 0.0001. AQP = aquaporin; CFA = complete Freund’s adjuvant; CHO = chinese hamster ovary cell line; DPI = days post immunization; EAE = experimental autoimmune encephalomyelitis; IgG = immunoglobulin G; Iso = isotype control; i.v. = intravenous; MOG = myelin oligodendrocyte glycoprotein.

To investigate disease manifestations in the spinal cord of MOG-IgG, AQP4-IgG, Iso-IgG EAE and sham immunized controls, we observed 18 animals for 11, 28 animals for 14–16 and 58 animals for 28–30 days after EAE induction or sham-immunization (Table 1). These time points correspond to presymptomatic, acute and chronic disease stages (Fig. 1). During the disease course, we evaluated clinical symptoms. After perfusion, CNS tissue was processed for comparative histopathological evaluations.

General and antibody-specific histopathological changes were compared between the three experimental disease model groups for each disease stage. The most relevant results are given in the respective figure within the manuscript, the other comparisons are provided in the Supplementary materials. Furthermore, within both antibody-augmented models, histopathological outcome of the different disease stages was compared.

### 2.2. Ethics approval and animal husbandry

Animal experiments were approved by the governmental authorities of the canton of Bern, Switzerland (BE134/16 and BE126/19) and performed in compliance with the ARRIVE guidelines (Animal Research: Reporting of In Vivo Experiments). Female C57Bl/6JRj wild type mice (Charles River Laboratories, Sulzfeld, Germany) were kept under standardized pathogen-free conditions including a stable light/dark cycle (12h:12 h) and access to food and water ad libitum. Experimental procedures were started after an acclimatization period of at least 7 days. Experiments were strictly randomized and analyzed in a blinded manner. Statistical planning assumed an α-error of 5% and 1-statistical power (β-error) of 20%.

### 2.3. Induction of antibody-augmented MOG_35–55_ EAE

Active EAE was induced in 8–10 weeks old mice. MOG peptide 35–55 (MOG_35–55_, 100 μg; Institute of Medical Immunology, Charité Berlin, Germany) emulsified in complete Freund’s adjuvant (CFA) was injected subcutaneously (s.c.) under anesthesia (50 mg/kg Ketamine; Ketanest^®^, Zurich, Switzerland and 1 mg/kg medetomidine; Dormitor^®^, Provet, Lyssach, Switzerland). After 30 min, mice were awakened by a s.c. antagonist injection (Atipamezol 1.25 mg/kg; Antisedan^®^, Provet), hydrated with 300 μl sodium chloride 0.9% solution and observed until fully recovered. Zero and two days post immunization (dpi), 200 ng pertussis toxin (List Biological Laboratories, Campbell, CA, USA) was injected intraperitoneally (i.p.).

At 10 dpi, animals were injected intravenously (i.v.) in the tail vein with 200 μg of murine monoclonal anti-MOG 8–18C5 IgG1 (2 mg/ml, purified by Aldevron, Freiburg, Germany) (Linington et al., 1988), human monoclonal recombinant anti-AQP4 IgG1 (rAb-53, 2 mg/ml) (Bennett et al., 2009) or a human monoclonal recombinant IgG1 isotype control (rAb-2B4 directed against measles virus nucleocapsid protein, 2 mg/ml) (Burgoon et al., 1999). Recombinant AQ4-IgG and Iso-IgG used herein were generated likewise from paired heavy and light chain sequences identified previously and tested for Fc effector function as well as target specificity (Bennett et al., 2009; Burgoon et al., 1999). Immunopathological effects should only result from different target specificity, but Iso-IgG is not expected to elicit additional pleiotropic effects.

At the respective endpoint, animals were euthanized by i.p. pentobarbital injection (150 mg/kg pentobarbital; Esconarkon^®^, Streuli Tiergesundheit AG, Uznach, Switzerland) and perfused with phosphate buffered saline followed by 4% paraformaldehyde (PFA) for further tissue processing.

### 2.4. Assessment of disease course

Disease severity was assessed as mobility impairment on a 10-point EAE scale: 0, asymptomatic; 1, reduced tone of tail; 2, limp tail, impaired righting; 3, absent righting; 4, gait ataxia; 5, mild paraparesis of hind limbs; 6, moderate paraparesis; 7, severe paraparesis or paraplegia; 8, tetraparesis; 9, moribund; 10, death (Bagnoud et al., 2020; Linker et al., 2002; Remlinger et al., 2022). A score of 7 on three consecutive days or a score of 8 or higher required early individual termination of the experiment for ethical reasons in two MOG-IgG EAE animals. As a conservative approach, these animals are continued in the clinical evaluation (EAE score) with the respective last score until end of experiment for all animals of the respective group. However, they were excluded from histopathological analyses if their individual endpoint reflected an earlier disease stage.

### 2.5. Histology, immunohistochemistry and immunofluorescence

Spinal cords were extracted and fixed in PFA for 24 h and embedded in paraffin and 5 μm thick transverse tissue sections were stained.

Demyelination was assessed after Luxol fast blue (LFB; Carl Roth, Arlesheim, Switzerland) / periodic acid Schiff (PAS; VWR International, Dietikon, Switzerland) staining. For immunohistochemistry (IHC), epitopes were unmasked with a Tris-EDTA buffer before blocking with fetal calf serum (Thermo Fisher Scientific, Waltham, MA, USA). IHC was performed for macrophages (rat anti-mouse Mac3, 0.3 μg/ml, BD Pharmingen, Heidelberg, Germany) and T cells (rat anti-human CD3, 10 μg/ml, Bio-Rad AbD Serotec, Puchheim, Germany) with a biotinylated secondary antibody (biotinylated rabbit anti-rat IgG, 2.5 μg/ml, Vector BA, Burlingame, CA, USA) and counterstaining with hematoxylin (VWR International, Dietikon, Switzerland).

Immunofluorescence staining served to visualize complement deposition with C5b-9 (mouse anti-human C5b-9, 10 μg/ml, Santa Cruz Biotechnology, Dallas, TX, USA), myelin integrity with myelin basic protein (rabbit anti-mouse MBP, 0.12 μg/ml, Abcam, Cambridge, UK), astrocytes with glial fibrillary acidic protein (chicken anti-mouse GFAP, 21.6 μg/ml, Abcam), AQP4 (rabbit anti-human AQP4, 4 μg/ml, Alomone Labs, Jerusalem, Israel), NK cells (goat anti-mouse NKp46/NCR1, 20 μg/ml, Novus Biologicals, Centennial, CO, USA) and NK cell activation with CD107a (rabbit anti-mouse LAMP1/CD107a, 2 μg/ml, Abcam).

Images were acquired with a slide scanner (Pannoramic 250 Flash III, 3DHISTECH, Budapest, Hungary) or a Nikon microscope equipped with epifluorescence and charge-coupled device (CCD) camera (Nikon Instruments Europe B.V., Egg, Switzerland) and evaluated using CaseViewer (3DHISTECH) or ImageJ (National Institute of Health, Bethesda, MD, USA), respectively.

### 2.6. Quantification of tissue assessments

All quantifications were performed in a blinded manner. In transverse spinal cord sections, demyelinated area was determined as percentage of total white matter after manual outlining of one lumbar and one thoracic cross-section. The number of T cells, macrophages, NK cells and NK cells expressing CD107a on the cell surface was manually quantified with CaseViewer in four regions of interest (ROI) of 100×100 μm within two lesions of the lumbar and thoracic spinal cord, each. For these quantifications, lesions are defined as demyelinating lesions with cellular infiltrates. GFAP and AQP4 fluorescence intensity were measured within the complete astrocytic lesions of two cervical, two thoracic and two lumbar cross-sections. Astrocytic lesion area was defined and outlined on images merged from GFAP, AQP4 and DAPI staining showing cell infiltration and incomplete GFAP and AQP4 immunofluorescence. Intensity values are expressed relative to a non-lesional area of the same cross-section or adjacent cross-section in case of extensive tissue injury. In the same lesions, astrocyte morphology was observed.

Localization and extent of demyelinating spinal cord lesions were evaluated from LFB/PAS and MBP stained images. Transverse spinal cord sections from each segment were analyzed: 2 cervical, 3 thoracic, 2 lumbar, 1 sacral. Lesions were classified as ventral, ventrolateral, lateral and dorsal localization. For quantification of lesion localization, all demyelinating lesions were counted per region per animal and expressed relative to the total amount of lesions counted within all available transverse spinal cord sections (*n* = 6–8) of the respective animal. For the graphic representation, demyelinating lesions in each region were scored according to their size (score 1–4) and expressed as percentage of all lesions at the respective region, averaged per segment in each group. Relative lesion size is indicated by a color scheme on the graph. MBP staining integrity (discontinuation of myelination and MBP granules) was evaluated within the ventral horn of all available transverse spinal cord sections per animal (*n* = 6–8).

### 2.7. Statistics

Statistical analysis was performed with Graph Pad 9 (Graph Pad Software INC., San Diego, CA, USA). Animal numbers indicated in the graphs result from pooled independent EAE experiments with the same experimental setup. Data are shown as mean ± standard error of the mean (SEM). Groups were compared by non-parametric tests with a 95% level of significance and correction for multiple comparison. Observed distributions were compared by chi-square (Chi^2^) test. Contingency tables comparing disease incidence were analyzed with Fisher’s exact test using SPSS (IBM SPSS Statistics Version 28.0.1.1, IBM, Armonk, NY, USA). Statistical tests performed are indicated in the figure legends. Levels of significance are indicated as follows: ns = not significant, *p < 0.05, ** *p* < 0.01, *** *p* < 0.001, **** *p* < 0.0001. For each dataset, ROUT outlier test was performed with *Q* = 1%.

### 2.8. Data availability

The datasets supporting the conclusions of this article are available to any qualified researcher on reasonable request.

## 3. Results

### 3.1. Differences in disease severity and incidence

Disease severity was higher in MOG-IgG compared to AQP4-IgG, Iso-IgG EAE and sham-immunized animals (mean area under the curve and 95% CIs: MOG-IgG 91.3 [81.0–101.5]; AQP4-IgG 48.8 [41.0–56.6]; Iso-IgG 43.3 [33.8–52.9], 2 independent experiments; Fig. 1A, B). Disease incidence was increased in both, MOG-IgG and AQP4-IgG EAE compared to Iso-IgG EAE (MOG-IgG and AQP4-IgG 100% vs. Iso-IgG 60%, Fisher’s exact test, *p* = 0.023, 2 independent experiments; Fig. 1A). Time to disease onset curves differ between MOG-IgG, AQP4-IgG and Iso-IgG EAE (Log-rank test, *p* = 0.0017; Fig. 1A) but were similar in a direct comparison of MOG-IgG and AQP4-IgG EAE (Log-rank test, *p* = 0.6913; Fig. 1B). Considering only diseased animals, onset of disease symptoms was similar in all EAE groups (mean: MOG-IgG 12 dpi, AQP4-IgG 13 dpi, Iso-IgG 14 dpi, Kruskal-Wallis test, *p* = 0.1152).

### 3.2. Histological correlates of inflammation and demyelination

**Fig. 2.**
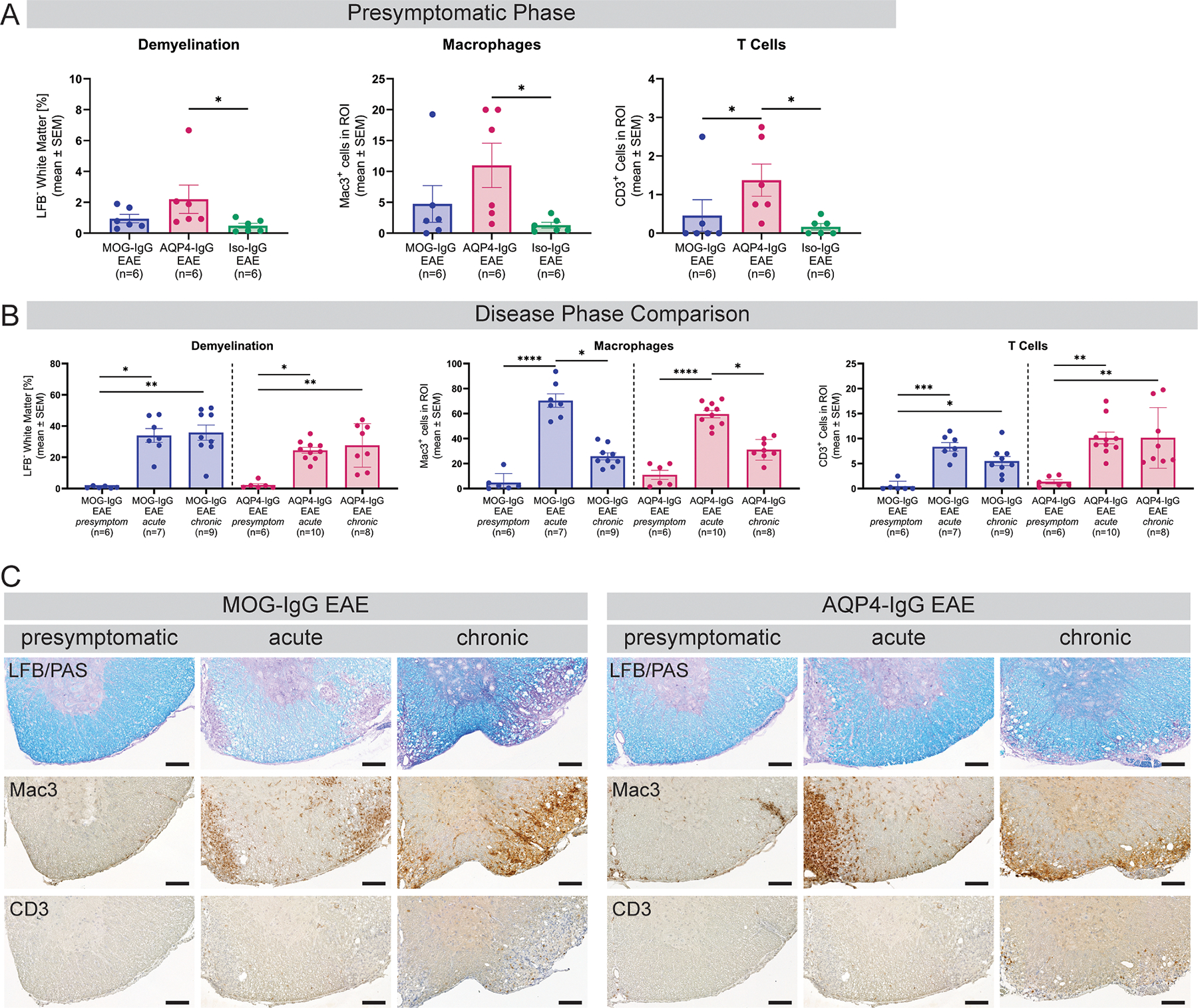
Quantification of demyelination and immune cell infiltration in the spinal cord. (A) Disease model comparison at presymptomatic disease phase: quantification of percentage of demyelination after LFB/PAS staining, macrophage infiltration after IHC for Mac3^+^ cells and T-cell infiltration after IHC for CD3^+^cells. One experiment. Kruskal-Wallis test. (B) Disease phase comparison of MOG-IgG and AQP4-IgG EAE animals for demyelination, macrophage infiltration and T cell infiltration. Three independent experiments. Kruskal-Wallis test. * *p* < 0.05, ** *p* < 0.01, *** *p* < 0.001, **** *p* < 0.0001. (C) Histologic representation of LFB/PAS, Mac3 and CD3 staining used for quantification at presymptomatic, acute and chronic disease phase. Scale bars = 100 μm. AQP = aquaporin; EAE = experimental autoimmune encephalomyelitis; IgG = immunoglobulin G; IHC = immunohistochemistry; Iso = isotype control; LFB = Luxol fast blue; MOG = myelin oligodendrocyte glycoprotein; PAS = periodic acid–Schiff; ROI = region of interest.

During the presymptomatic disease phase, T cell infiltration was higher in AQP4-IgG than MOG-IgG or Iso-IgG EAE (Fig. 2A). Macrophage infiltration and demyelination were higher in presymptomatic AQP4-IgG compared to Iso-IgG, but not MOG-IgG EAE (Fig. 2A). Neither differences were found between the three groups at acute or chronic disease stages (Figure A.1A, B). Over the disease course, macrophages were increased in both MOG-IgG and AQP4-IgG EAE at acute disease stage compared to presymptomatic and chronic stage. T cell infiltration and demyelination were increased at acute and chronic compared to presymptomatic disease stage in both antibody-mediated models (Fig. 2B, C).

### 3.3. Natural killer cell involvement and complement deposition

**Fig. 3.**
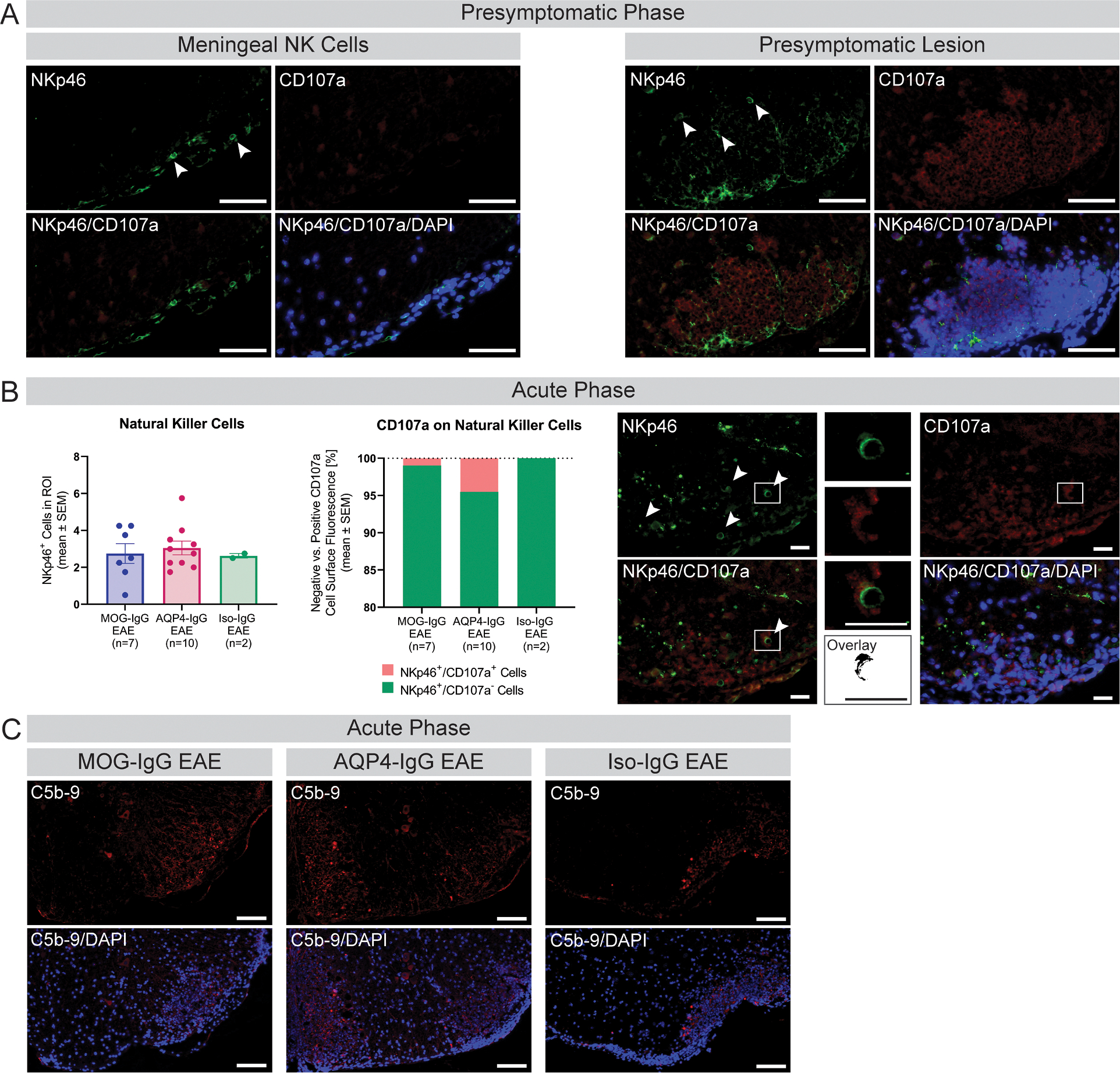
Investigation of NK cell involvement and complement deposition to assess ADCC and CDC in the spinal cord. (A) Histologic representation of NKp46 and CD107a staining (DAPI counterstain) at presymptomatic disease phase with arrows indicating NKp46 positive cells within meninges (left, Iso-IgG EAE animal) and lesions present before onset of clinical disease symptoms (right, MOG-IgG EAE animal). Scale bars = 50 μm. (B) Disease model comparison of number of NKp46 positive cells (NK cells) and percentage of NKp46 positive cells with CD107a negative (green) and positive (red) staining at the cell surface (activated NK cells) in spinal cord lesions at acute disease phase. Two independent experiments. Kruskal-Wallis test. *p*=ns. Histologic representation of NKp46 and CD107a IF staining (DAPI counterstain) at acute disease phase (AQP4-IgG EAE animal). Arrows showing NKp46 positive (upper left) and NKp46/CD107a double positive (lower left) cells, respectively. Higher magnification of areas boxed showing a NKp46/CD107a double positive cell with overlay analysis performed in ImageJ. Scale bars = 20 μm. (C) Histologic investigation of complement deposition in MOG-IgG-, AQP4-IgG-augmented and Iso-IgG EAE at acute disease phase. Scale bars = 100 μm. ADCC = antibody-dependent cellular cytotoxicity; AQP = aquaporin; CDC = complement-dependent cytotoxicity; DAPI = 49,6-diamidino-2-phenylindole; EAE = experimental autoimmune encephalomyelitis; IgG = immunoglobulin G; IF = immunofluorescence; Iso = isotype control; MOG = myelin oligodendrocyte glycoprotein; ROI = region of interest.

At presymptomatic disease stage, NK cells were mostly found in the meninges and not the CNS parenchyma in all EAE groups (Fig. 3A). Four animals showed inflammatory lesion formation before onset of disease symptoms (two MOG-IgG EAE and two AQP4-IgG EAE). Here, NK cells were also present in the tissue parenchyma (Fig. 3A). During acute phase, NK cells were found in all diseased animals with few NK cells showing increased CD107a positivity at the cell surface (Fig. 3B). Sparse complement deposition was found in diseased animals at acute but not presymptomatic stage regardless of antibody augmentation model (Fig. 3C, Figure A.1).

### 3.4. GFAP and AQP4 fluorescence intensity

**Fig. 4.**
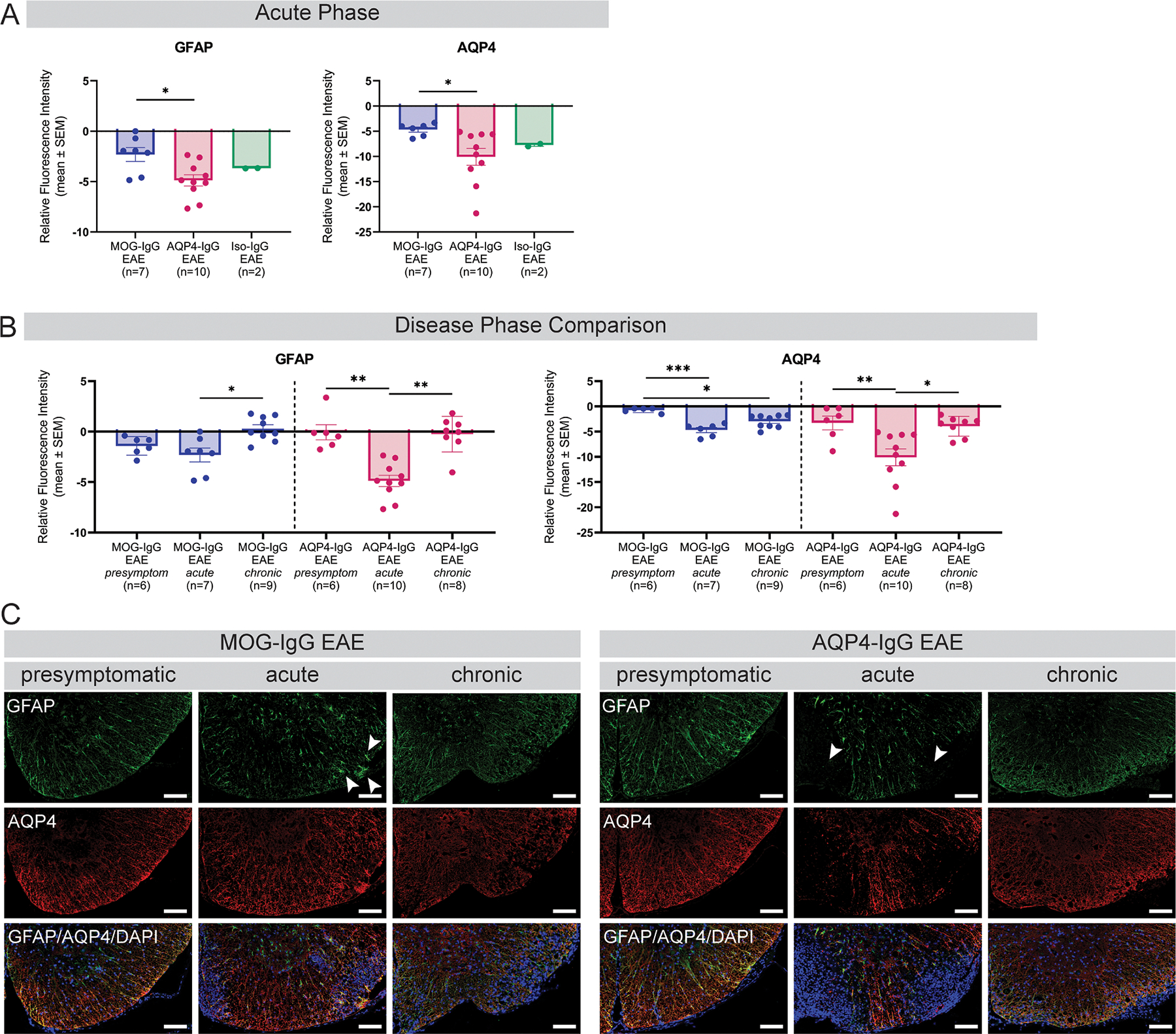
Investigation of targeted and bystander injury to astrocytes and AQP4 water channels. (A) Disease model comparison of GFAP and AQP4 fluorescence intensity, respectively, at acute disease phase within spinal cord lesions normalized to fluorescence in a non-lesion area. Two independent experiments. Kruskal-Wallis test. (B) Disease phase comparison of GFAP and AQP4 fluorescence intensity in MOG-IgG and AQP4-IgG EAE animals separately. Three independent experiments. Kruskal-Wallis test. * *p* < 0.05, ** *p* < 0.01, *** *p* < 0.001. (C) Histologic characterization of GFAP and AQP4 fluorescence counterstained with DAPI at presymptomatic, acute and chronic disease phase. Arrows indicate presence of GFAP staining in lesions of MOG-IgG EAE with swollen astrocytes. In AQP4-IgG EAE animals, arrows point out complete GFAP loss in the lesion center. Residual GFAP staining at the border of the lesion indicates dystrophic astrocytes. Scale bars = 100 μm. AQP = aquaporin; DAPI = 49,6-diamidino-2-phenylindole; EAE = experimental autoimmune encephalomyelitis; GFAP = glial fibrillary acidic protein; IgG = immunoglobulin G; IF = immunofluorescence; Iso = isotype control; MOG = myelin oligodendrocyte glycoprotein.

Quantification of GFAP as a marker of astrocytes and AQP4 immunofluorescence within astrocytic spinal cord lesions showed a reduction of both GFAP and AQP4 at acute disease stage in AQP4-IgG compared to MOG-IgG EAE (Fig. 4A). No differences in immunofluorescence between the EAE groups were detected at presymptomatic or chronic disease stage (Figure A.2A, B). Within the MOG-IgG EAE group, GFAP immunofluorescence was reduced at acute compared to chronic disease stage and AQP4 immunofluorescence was lower at acute and chronic disease stage compared to presymptomatic stage (Fig. 4B). AQP4-IgG EAE animals showed reduced immunofluorescence of GFAP and AQP4 at acute compared to presymptomatic and chronic disease stage (Fig. 4B). Astrocyte morphology seemed different in AQP4-IgG compared to MOG-IgG EAE at acute and chronic disease stage: In MOG-IgG EAE animals, astrocytes kept their general structure, even when reduced in number, with changes in morphology (mainly swelling), whereas AQP4-IgG augmentation seemed to disintegrate astrocytes (e.g. after beading/clasmatodendrosis) with only little residual GFAP staining in astrocytic lesions (Fig. 4C, Figure A.2).

### 3.5. Demyelinating lesion localization in different models

**Fig. 5.**
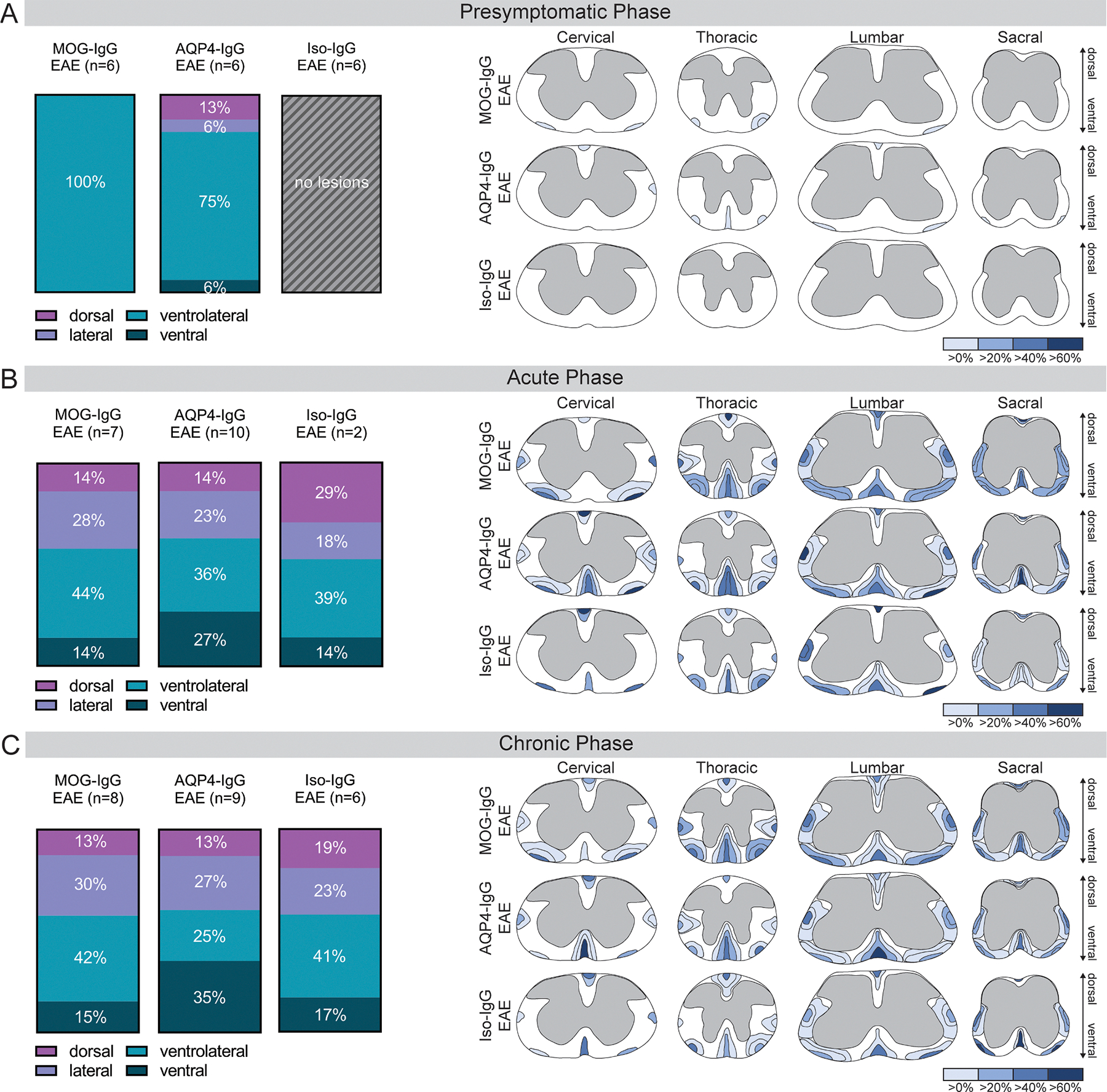
Localization of inflammatory lesions at different disease phases. Quantification and graphic representation of relative localization of inflammatory, demyelinating lesions in the spinal cord at (A) presymtomatic, (B) acute and (C) chronic disease phase. Left panel: Percentage of the respective lesion localization in relation to all lesions. Disease model comparison by Chi^2^ test. (A) One experiment, 6 animals per group analyzed (2 MOG-IgG and 2 AQP4-IgG animal with lesions), *p*=ns. (B) Two independent experiments, *p* = 0.0089. (C) Two independent experiments, *p* = 0.0052. Right panel: Graphic representation of the relative size and proportion of white matter lesions at the investigated sites in the cervical, thoracic, lumbar, and sacral segments of the spinal cord. Proportion of the extent of lesions at the respective localizations are indicated with a color scheme (very light blue: 1–20%, light blue: 21–40%, blue: 41–60%, dark blue: 61–100% of lesions extend this broadly). ANOVA = analysis of variance; AQP = aquaporin; EAE = experimental autoimmune encephalomyelitis; IgG = immunoglobulin G; Iso = isotype control; MOG = myelin oligodendrocyte glycoprotein.

Comparison of proportion and size of demyelinating spinal cord lesions at preferentially affected sites (ventral, ventrolateral, lateral and dorsal) at presymptomatic disease stage showed few small lesions without differential distribution between MOG-IgG and AQP4-IgG EAE. Lesion formation at this stage was not detected in Iso-IgG EAE (Fig. 5A). Lesions at acute and chronic disease stage were located differently with a predisposition for ventral lesions in AQP4-IgG EAE animals and ventrolateral lesions in MOG-IgG and Iso-IgG EAE (acute: Chi^2^ test, *p* = 0.0089; Fig. 5B; chronic: Chi^2^ test, *p* = 0.0052; Fig. 5C).

### 3.6. Deep demyelinating white matter lesions and associated gray matter demyelination (GMD)

**Fig. 6.**
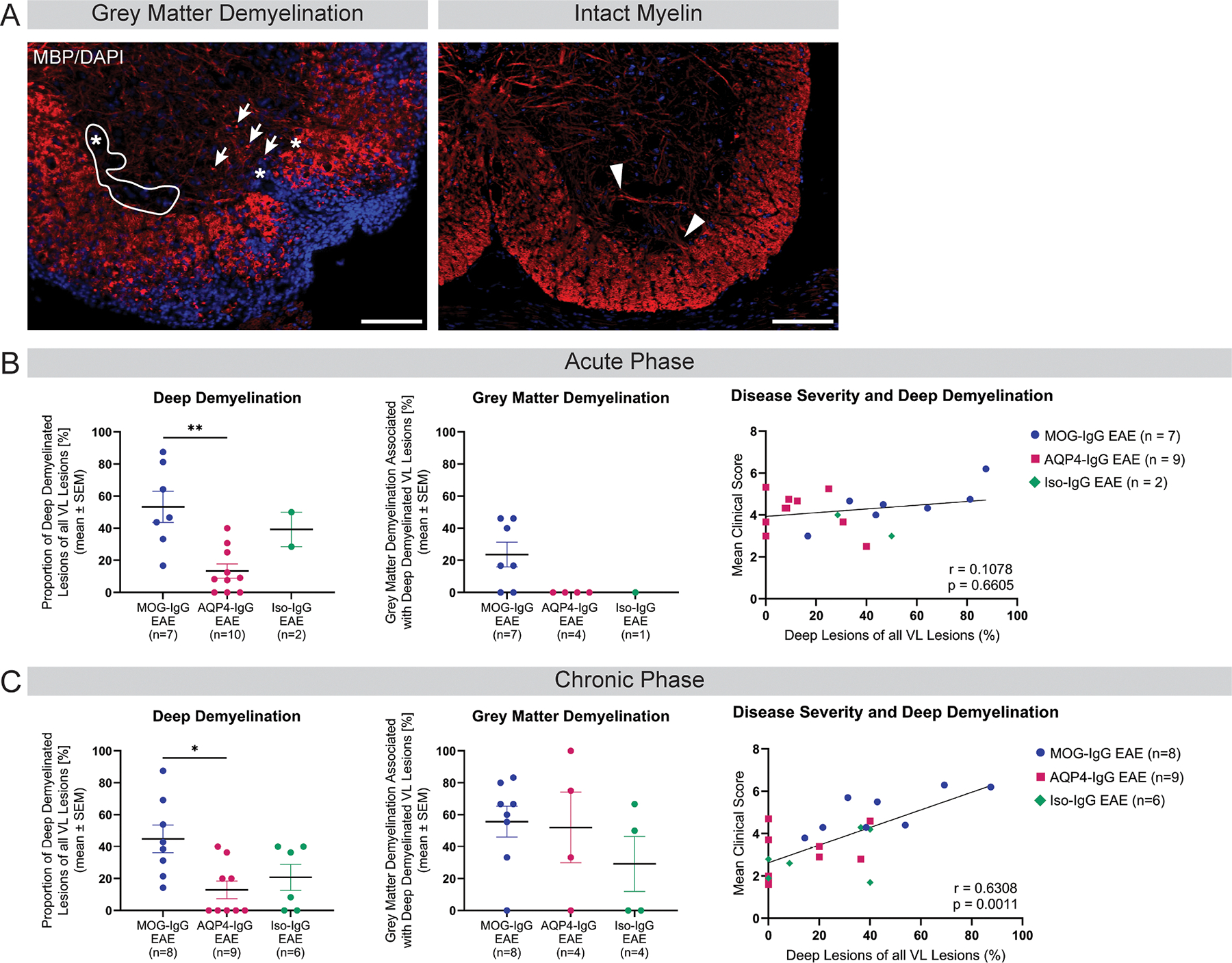
Deep white matter demyelination and associated gray matter involvement (A) Representative MBP IF staining of a deep ventrolateral demyelinated lesion with loss of fiber integrity (asterix, discontinuation of myelinated fibres between white and gray matter) and MBP granule deposition (arrows), left. Representative MBP IF staining of a sham-immunized control animal showing intact myelinated fiber integrity (triangle), right. Scale Bars = 100 μm. (B) Acute and (C) chronic disease phase quantification of deep VL demyelinating lesions extending from meninges to gray matter border of all VL lesions per animal (panel 1). Evaluation of proportion of lesions with signs of gray matter demyelination associated with deep ventrolateral lesions (panel 2). Kruskal-Wallis test, * *p* < 0.05, ** *p* < 0.01. Correlation analysis of mean clinical score and proportion of deep VL lesions (Panel 3). Spearman correlation. Correlation coefficient r and p-value are indicated on graph. AQP = aquaporin; DAPI = 49,6-diamidino-2-phenylindole; EAE = experimental autoimmune encephalomyelitis; IgG = immunoglobulin G; IF = immunofluorescence; Iso = isotype control; MBP = myelin basic protein; MOG = myelin oligodendrocyte glycoprotein; VL = ventrolateral.

In the quantification of ventrolateral demyelinating lesions that extend from the meninges to the gray matter (i.e. deep lesions), ventral horns associated with deep lesions were further evaluated for GMD defined as loss of MBP-positive fiber integrity and deposition of MBP-positive granules in the ventral horn (Fig. 6A). No deep lesions or signs of GMD were detected before onset of disease symptoms or in sham-immunized controls. MOG-IgG EAE animals demonstrated more deep ventrolateral lesions than AQP4-IgG or Iso-IgG EAE animals at acute and chronic disease stage (Fig. 6B, C). A similar proportion of these deep lesions were associated with GMD in the ventral horn of all EAE groups at acute and chronic disease stage (Fig. 6B, C). At chronic but not acute stage, proportion of deep ventrolateral lesions is higher in more severely diseased animals (Spearman correlation coefficient chronic: *r* = 0.63, *p* = 0.0011, acute: *r* = 0.11, *p* = 0.66; Fig. 6B, C).

## 4. Discussion

In a direct comparison of different antibody-mediated murine models of CNS demyelination in a parallel experimental approach, we observed differences in spinal cord immunopathology between models and phases of the disease. Even though only MOG-IgG administration increased disease severity, both MOG-IgG and AQP4-IgG administration led to a higher disease incidence than the antibody control group (using a monoclonal IgG directed against a non-CNS-specific antigen, measles virus nucleocapsid protein (Bennett et al., 2009; Burgoon et al., 1999; Phuan et al., 2012; Soltys et al., 2019)). Although it was already shown that MOG-IgG administration augments disease severity (Lassmann et al., 1988; Linington et al., 1988) in contrast to AQP4-IgG (Saini et al., 2013), the differences in incidence may suggest different pathophysiological mechanisms in AQP4-IgG EAE. Indeed, before onset of mobility impairment, we observed more pronounced classic histopathologic correlates of neuroinflammation in AQP4-IgG EAE compared to Iso-IgG EAE. In line with previous data (Saini et al., 2013), this early neuroinflammation does not translate into an increased disease severity later on. At later time points, the disease models were similarly affected by immune cell infiltration and demyelination. Disease hallmarks over time were comparable for MOG- and AQP4-IgG EAE. Demyelination and T cell infiltration increased from presymptomatic to acute phase and remained at a high level. Whereas macrophages also heavily infiltrated the spinal cord tissue from presymptomatic to acute phase, their number decreased again towards the chronic phase, reflecting the dynamics in disease severity. In AQP4-IgG-mediated pathology, high neutrophil infiltration favoring the interaction of CNS antigen-specific T cells with the BBB could promote earlier lesion expansion (Aubé et al., 2014; Bradl et al., 2009; Saadoun et al., 2012).

Complement-dependent (CDC) and -independent mechanisms (ADCC) of tissue destruction have been associated with MOG-IgG and AQP4-IgG-excacerbated autoantigen destruction in animal models (Bennett et al., 2009; Duan et al., 2019; Phuan et al., 2012; Saadoun et al., 2014; Spadaro et al., 2018). In our study, we showed both complement deposition and infiltration of NK cells in the context of lesion formation in all diseased animals regardless of antibody specificity. While animals without clinical disease symptoms at acute stage were devoid of NK cells and complement deposition, we showed NK cells in the meninges of animals before onset of disease symptoms. Even though surface mobilization of CD107a is used as a proxy of NK-mediated cytotoxic activity (Vincent et al., 2008), such investigations are usually performed in vitro using flow cytometry. ADCC induction in vivo has been shown for AQP4-IgG rAb53 (Ratelade et al., 2013; Ratelade et al., 2012). One study using MOG-IgG 8–18C5 is suggestive of ADCC but does not entirely exclude antibody-dependent cellular phagocytosis (ADCP) as an alternative or additional mechanism (Piddlesden et al., 1991). In our experimental setup, we attempted to approximate ADCC as a mechanism involved in tissue destruction with immunofluorescence staining of CD107a on the surface of NK cells. A possible involvement of activated macrophages or neutrophils in ADCC has not further been analyzed in the present study. However, as recently novel methods have been described to detect macrophage involvement, this might be relevant for future investigations (Uccellini et al., 2021). In addition, Fc blocking, protease inhibition and microglial depletion individually or in concert might further elucidate the role of ADCC and ADCP in these model systems.

Investigation of AQP4 and astrocytes as targets of AQP4-IgG-mediated autoimmunity showed differences between AQP4-IgG and MOG-IgG EAE at acute disease stage. No differences were found comparing the Iso-IgG EAE with the caveat of a low number of diseased animals in that group. GFAP fluorescence recovered towards the chronic disease phase in MOG-IgG EAE. Yet, AQP4 immunofluorescence remained reduced compared to a presymptomatic disease phase. This indicates acute bystander astrocyte gliosis in MOG-IgG EAE, which improves in the chronic disease phase. However, recovery of astrocytic AQP4 is delayed, possibly indicating continued internalization of AQP4 due to the inflammatory environment and proinflammatory astrocyte changes not displayed by loss of GFAP (Chen et al., 2020). In contrast, GFAP decreased in acute disease stage of AQP4-IgG EAE with restoration in the chronic disease phase. Here, the dynamics of AQP4 loss and recovery behave analogous to GFAP suggesting that loss of AQP4 induced by AQP4-IgG is the main driver of astrocyte damage in this model (Hinson et al., 2017). These quantitative observations are further supported by qualitative analyses of astrocytic spinal cord lesions as astrocytes in MOG-IgG EAE lesions were largely recognizable as entire cells despite distorted morphology. On the other hand, AQP4-IgG augmentation seemed to be more destructive with only residual GFAP positivity in the lesions indicating dystrophic astrocytes (small GFAP-positive residues possibly indicating beading/clasmatodendrosis). This is in line with observed events in lesions of MOG-IgG-seropositive patients showing active astrogliosis and little dystrophic astrocytes (Höftberger et al., 2020; Lopez et al., 2021) in contrast to AQP4-IgG-seropositive patients with dystrophic astrocytes and striking loss of AQP4 in active lesions (Popescu et al., 2015). Contrastingly, these human studies did not show loss of AQP4 within lesions of MOG-IgG-seropositive patients or persisting AQP4 loss in AQP4-IgG seropositive patients. This disparity may indicate greater bystander injury mediated by the MOG-IgG 8–18C5 used in our model system that may have a different target and propensity for complement activity demonstrated *ex vivo* (Peschl et al., 2017). Even though there was no difference in AQP4 immunofluorescence from presymptomatic to chronic disease phase in our AQP4-IgG EAE model, AQP4 was decreased at chronic disease stage compared to sham-immunized controls.

Different patterns of demyelinating lesion distribution within the brain and spinal cord have been immensely valuable for the differentiation of MOGAD, NMOSD and MS in clinical practice (Jarius et al., 2018; Juryńczyk et al., 2017; Wingerchuk et al., 2015). Our direct comparison in animal models suggests a predilection for ventral neuroinflammatory lesions associated with AQP4-IgG and ventrolateral lesions in purely MOG-targeted autoimmunity regardless of MOG-IgG-augmentation. Although we could not statistically determine differences of the few lesions in the presymptomatic phase, it is rather striking that all lesions found in MOG-IgG EAE animals were located ventrolateral whereas lesions in AQP4-IgG EAE animals already arose at all investigated locations. Moreover, Iso-IgG EAE animals had no lesions this early. Differences in lesion site and size may be explained by an imbalance of encephalitogenic T cells activated by immunization and CNS-specific antibodies (Lassmann et al., 1988). Antigen specificity of T cells may not only influence topographic differences of the CNS lesions but also the extent of inflammation and macrophage activation (Berger et al., 1997).

Even though deep VL demyelinating white matter lesions were more frequent in MOG-IgG EAE compared to AQP4-IgG or Iso-IgG, the proportion of these lesions with associated GMD was similar in all groups. Thus, ventral horn GMD may rather be a consequence of VL white matter lesion depth in a *per continuitatem* process than a differential mechanism of different model systems. The presence of GMD in murine MOG_35–55_ EAE is in line with earlier studies also showing glial activation and inflammatory changes in the spinal cord ventral horn (Wu et al., 2008). However, GMD seems not to be a predominant feature of murine MOG_35–55_ EAE, but was well characterized in a subclinical MOG_35–55_ EAE rat model with targeted induction of cortical inflammation and demyelination (Merkler et al., 2006).

These data also call attention to a limitation of our model approach that is the use of MOG peptide-induced EAE not only breaching the BBB and providing an underlying CNS inflammation/demyelination, but creating a mainly T cell driven disease itself. Earlier experiments demonstrated that an intact BBB and even a PTX-permeabilized BBB did not allow lesion formation and loss of AQP4 in animals receiving NMO-IgG intravenously or intraperitoneally (Bradl et al., 2009; Ratelade et al., 2011). Studies using a similar approach as ours have well established the necessity of an underlying CNS inflammation for passively administered antibodies to reach the CNS and bind target structures (Kinoshita et al., 2009; Linington et al., 1988; Remlinger et al., 2022). Other murine NMOSD models attempting immunization with an AQP4 peptide alone or in combination with adoptive transfer of AQP4-specific T cell could not sufficiently reproduce histopathological hallmarks (Duan and Verkman, 2020; Kalluri et al., 2011). Immunization of AQP4-deficient, but not wildtype mice with AQP4 peptide 135–153 or 201–220 elicits a T cell response (Sagan et al., 2016). Transfer of these AQP4-specific Th17 T cells isolated from AQP4-deficient donor mice induced paralysis in naïve WT mice, which was associated with leptomeningeal inflammation of the spinal cord and optic nerves (Sagan et al., 2017). Even though MOG peptide immunization represents a different artificial approach part to disease development in our set-up, many experimental studies using a similar approach have contributed immensely the understanding of pathophysiological mechanisms (Bennett et al., 2009; Saini et al., 2013). As our goal was mainly to study antibody-associated features, we have chosen to start from a common initial disease induction, which can also be favored from a 3R perspective avoiding breeding of another mouse strain and generating transferable cells back to naïve mice. This animal model in combination with disease-specific pathogenic antibodies replicates important features of the human diseases NMOSD and MOGAD with a high incidence of disease and reproducible disease course (Bennett et al., 2009; Bradl et al., 2009; Kinoshita et al., 2009; Lassmann et al., 1988; Linington et al., 1988; Saini et al., 2013). We did not further compare Iso-IgG EAE to naïve MOG_35–55_ EAE. The isotype control antibody used in this study is a monoclonal IgG specific for the non-CNS-specific antigen measles virus nucleocapsid protein (Burgoon et al., 1999). Thus, it should not interfere with EAE unlike a mixture of polyclonal IgG. After intrathecal or intravenous injection into EAE animals, this Iso-IgG did not alter or produce additional CNS immunopathology (Bennett et al., 2009). In a direct comparison with NMOSD patient-derived antibodies, this Iso-IgG did not disrupt BBB function (Shimizu et al., 2017). Mice showed normal cortical vasculature, blood flow and BBB integrity after in utero exposure to this Iso-IgG (Mader et al., 2022). No effect on AQP4-expressing CHO cells was observed in vitro (Ratelade et al., 2012).

## 5. Conclusions

Our parallel experimental approach using animal models of MOG-IgG-, AQP4-IgG-augmented and unspecific IgG CNS demyelination compares important pathophysiological aspects mimicking parts of the human diseases MOGAD, NMOSD and MS. The described models allow further analyses of pathological features and disease processes associated with antibodies against MOG or AQP4. The comparative characterization may facilitate the investigation of therapeutic interventions in regard to their effect on different key players in lesion formation and disease progression.

## Supplementary Material

Supplement

## Figures and Tables

**Table 1:** Study overview.

Animal Model	Immunization (0 dpi)	Augmentation (10 dpi)	Perfusion Presymptomatic (11 dpi)	Perfusion Acute (14–16 dpi)	Perfusion Chronic (28–30 dpi)

MOG-IgG EAE	MOG/CFA	MOG-IgG	*N* = 6 (0)	*N* = 11 (8)	*N* = 20 (19)
AQP4-IgG EAE	MOG/CFA	AQP4-IgG	*N* = 6 (0)	*N* = 11 (10)	*N* = 19 (17)
Iso-IgG EAE	MOG/CFA	Iso-IgG	*N* = 6 (0)	*N* = 6 (2)	*N* = 10 (6)
Sham Immunization	CFA	Iso-IgG			*N* = 9 (0)

Overview of the animals immunized and administered with the respective antibodies for analysis at the specified disease phases. At each disease phase, the number of immunized animals is indicated with the number of animals showing clinical symptoms in brackets.

AQP = aquaporin; CFA = complete Freund’s adjuvant; DPI = days post immunization; EAE = experimental autoimmune encephalomyelitis; Iso = isotype control; MOG = myelin oligodendrocyte glycoprotein.

## Data Availability

The datasets supporting the conclusions of this article will be made available in the Bern Open Repository and Information System (BORIS).

## Abbreviations:

95CI: 95% confidence interval
ADCC: antibody-dependent cellular cytotoxicity
ANOVA: analysis of variance
AUC: area under curve
AQP4: aquaporin 4
BBB: blood brain barrier
CCD: charge-coupled device
c/d: cycles per degree
CDC: complement-dependent cytotoxicity
CFA: complete Freund’s adjuvant
CNS: central nervous system
dpi: days post immunization
DAPI: 49,6-diamidino-2-phenylindole
EAE: experimental autoimmune encephalomyelitis
IgG: immunoglobulin G
IF: immunofluorescence
GFAP: glial fibrillary acid protein
GMD: gray matter demyelination
IHC: immunohistochemistry
i.p.: intraperitoneal
Iso: isotype control
i.v.: intravenous
LFB: Luxol fast blue
MOG: myelin oligodendrocyte glycoprotein
MOGAD: myelin oligodendrocyte glycoprotein antibody-associated disorder
MS: multiple sclerosis
NK: natural killer
NMOSD: neuromyelitis optica spectrum disorder
PAS: periodic acid–Schiff
PFA: paraformaldehyde
rAb: recombinant antibody
RGC: retinal ganglion cells
ROI: region of interest
s.c.: subcutaneous
SEM: standard error of mean
