# Supplementary material for: Modelling MOG antibody-associated disorder and neuromyelitis optica spectrum disorder in animal models: Spinal cord manifestations
Source: Mult Scler Relat Disord. Author manuscript; Available in PMC 2025 Feb 4. (PMC11792092; doi:10.1016/j.msard.2023.104892)
Supplement: Supplement [file NIHMS2047034-supplement-Supplement.docx]

# Appendix

# Supplementary Figures and Figure Legends

### Figure A.1. Quantification of demyelination and immune cell infiltration in the spinal cord at acute and chronic disease phase.


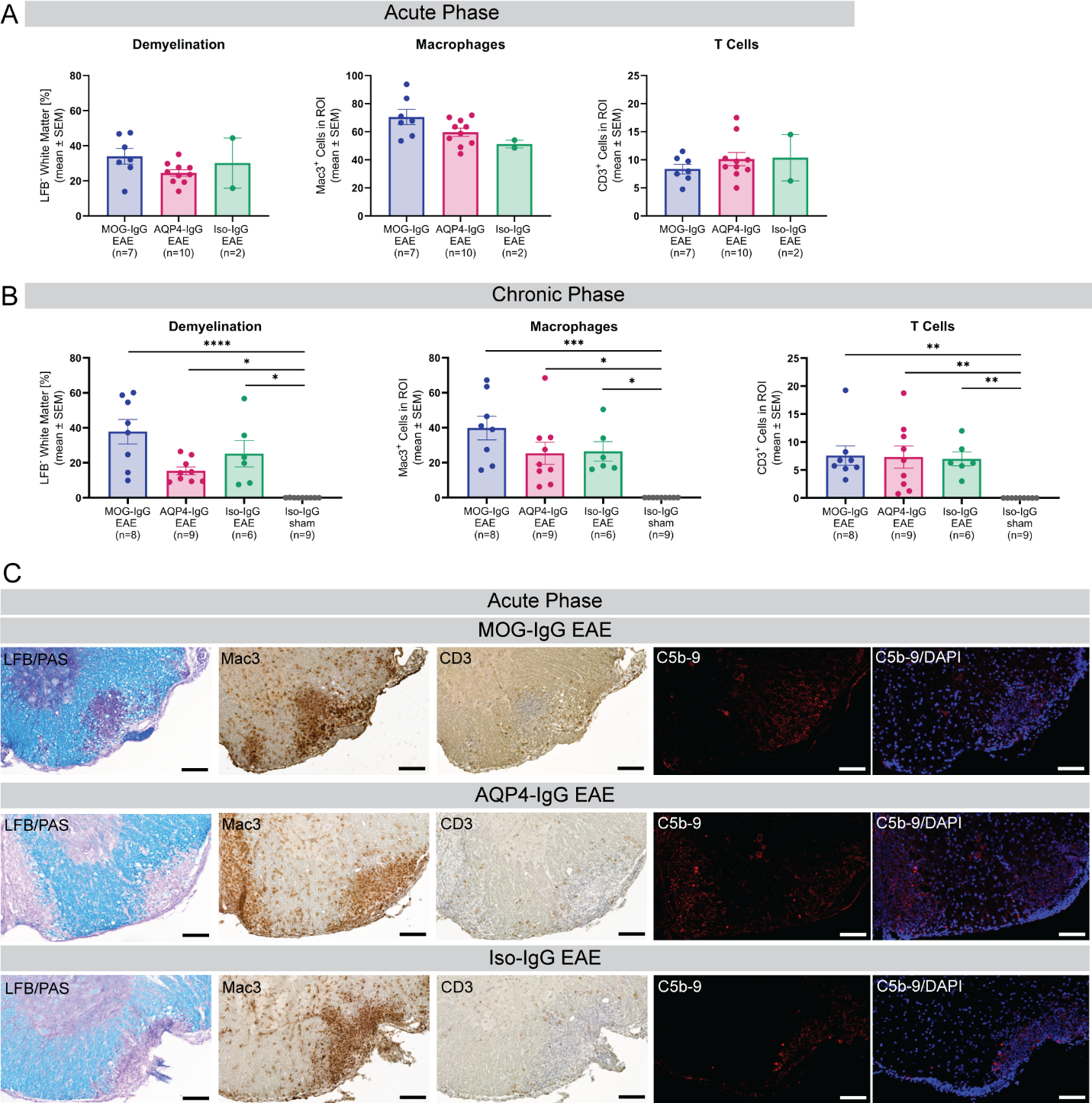


Disease model comparison at (A) acute and (B) chronic disease phase: Quantification of percentage of demyelination after LFB/PAS staining, macrophage infiltration after IHC for Mac3+ cells and T-cell infiltration after IHC for CD3+ cells. Two independent experiments per disease phase. Kruskal-Wallis test. *p<0.05, **p<0.01, ***p<0.001. (C) Direct histologic comparison of LFB/PAS, Mac3, CD3 and complement staining at acute disease phase. Scale bars = 100 µm.

AQP = aquaporin; CFA = complete Freund’s adjuvant; IgG = immunoglobulin G; Iso = isotype control; IHC = immunohistochemistry; LFB = Luxol fast blue; MOG = myelin oligodendrocyte glycoprotein; PAS = periodic acid–Schiff; ROI = region of interest.

### Figure A.2. Investigation of targeted and bystander injury to astrocytes and AQP4 water channel at presymptomatic and chronic disease phase.


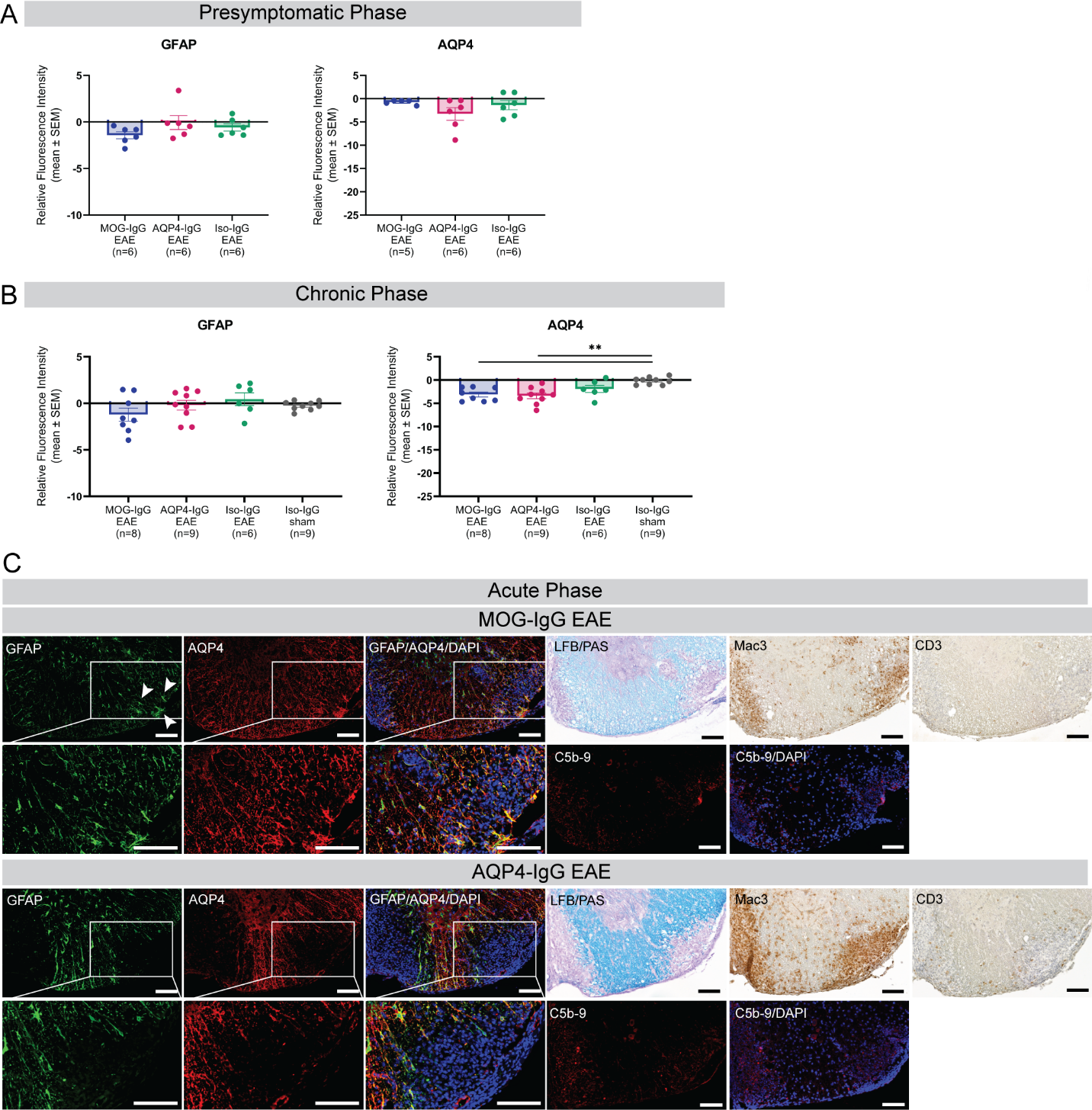


Disease model comparison of GFAP and AQP4 fluorescence intensity, respectively, at (A) presymptomatic and (B) chronic disease phase within spinal cord lesions normalized to fluorescence in a non-lesion area. Presymptomatic: one experiment, chronic: two independent experiments per disease phase. Kruskal-Wallis test. **p<0.01. (C) Higher magnification of areas boxed on GFAP and AQP4 staining images and comparison to LFB/PAS, Mac3, CD3 and complement staining at acute disease phase. Scale bars = 100 µm.

AQP = aquaporin; CFA = complete Freund’s adjuvant; GFAP = glial fibrillary acidic protein; IgG = immunoglobulin G; IF = immunofluorescence; Iso = isotype control; MOG = myelin oligodendrocyte glycoprotein.
